# Ramadan Fasting During the COVID-19 Pandemic; Observance of Health, Nutrition and Exercise Criteria for Improving the Immune System

**DOI:** 10.3389/fnut.2020.570235

**Published:** 2021-01-13

**Authors:** Majid Taati Moghadam, Behzad Taati, Seyed Mojtaba Paydar Ardakani, Katsuhiko Suzuki

**Affiliations:** ^1^Department of Microbiology, School of Medicine, Iran University of Medical Sciences, Tehran, Iran; ^2^Student Research Committee, Iran University of Medical Sciences, Tehran, Iran; ^3^Department of Exercise Physiology, Faculty of Sports Sciences, University of Guilan, Rasht, Iran; ^4^Department of Sport Sciences, Faculty of Humanities and Social Sciences, Ardakan University, Yazd, Iran; ^5^Faculty of Sport Sciences, Waseda University, Tokorozawa, Japan

**Keywords:** coronavirus disease 2019, exercise training, functional foods, inflammatory responses, muslim athletes, lifestyle modifications

## Abstract

Fasting is one of the religious rituals of Muslims worldwide who refrain from eating foods and liquids every year during Ramadan. This year (2020), Ramadan is very different from previous years due to the outbreak of a terrible microscopic giant called coronavirus disease 2019 (COVID-19). The pandemic COVID-19 has made Ramadan very important this year because the virus has infected millions of people around the world and killed thousands, especially people with immunodeficiency. In dealing with COVID-19, maintaining good hygiene and supporting the immune system are effective, preventive approaches. Moderate exercise training and proper nutrition are the most important factors to support immune function. Lack of facilities, poor health and many traditions that lead to public community gatherings have made many Islamic countries susceptible to this dangerous virus. In such an unprecedented situation, there are many Muslims who doubt whether they can fast or not. Therefore, the proposal of usable exercise programs and effective nutritional strategies is imperative. In this study, we will look at the proposed health effects of fasting and its impact on the immune system, the effects of Ramadan intermittent fasting on resting values and responses of immunological/antioxidant biomarkers in elite and recreational athletes, together with the important health, nutrition, and exercise advice that fasting people need to follow in the event of a COVID-19 outbreak.

## Introduction

Muslims were estimated to make up about one-fourth (1.6 billion) of the world's population, and Islam is the second largest religion after Christianity (1). One of the religious rituals of healthy adult Muslims is fasting from sunrise to sunset in Ramadan (during 29 or 30 days). Fasting means avoiding drinking and eating over time periods that vary from 13 to 18 h a day, depending on the season (2). Since human coronaviruses have been identified around the world, they have been identified as mild human pathogens. The most well-known coronaviruses are human pathogens, including Middle East Respiratory Syndrome (MERS-CoV) and Acute Respiratory Syndrome (SARS-CoV), which have killed large numbers of people in Asia and the Middle East (3). The MERS-CoV virus was first reported in 2012 in Saudi Arabia from a sputum sample of a patient with pneumonia (4). However, in 2019, coronavirus developed a more severe respiratory disease in lung cells, caused by a novel coronavirus called COVID-19. It has been several months since COVID-19 spread from China, but in such a short time a pandemic has occurred due to its very high spreading capability (5). Humans have failed to fight this deadly virus successfully, and about 6 million people worldwide have been infected with the virus, thousands of whom have died. Older people with underlying health problems, and/or immunodeficiency, are more likely to have a poor outcome when they are infected with the virus (5, 6). So far, the exact number of deaths from COVID-19 is not known, due to the actual number of patients not having been identified, but previous studies have reported that ~2. 84% of patients die and at least 10% show symptoms (7).

**Figure 1:**
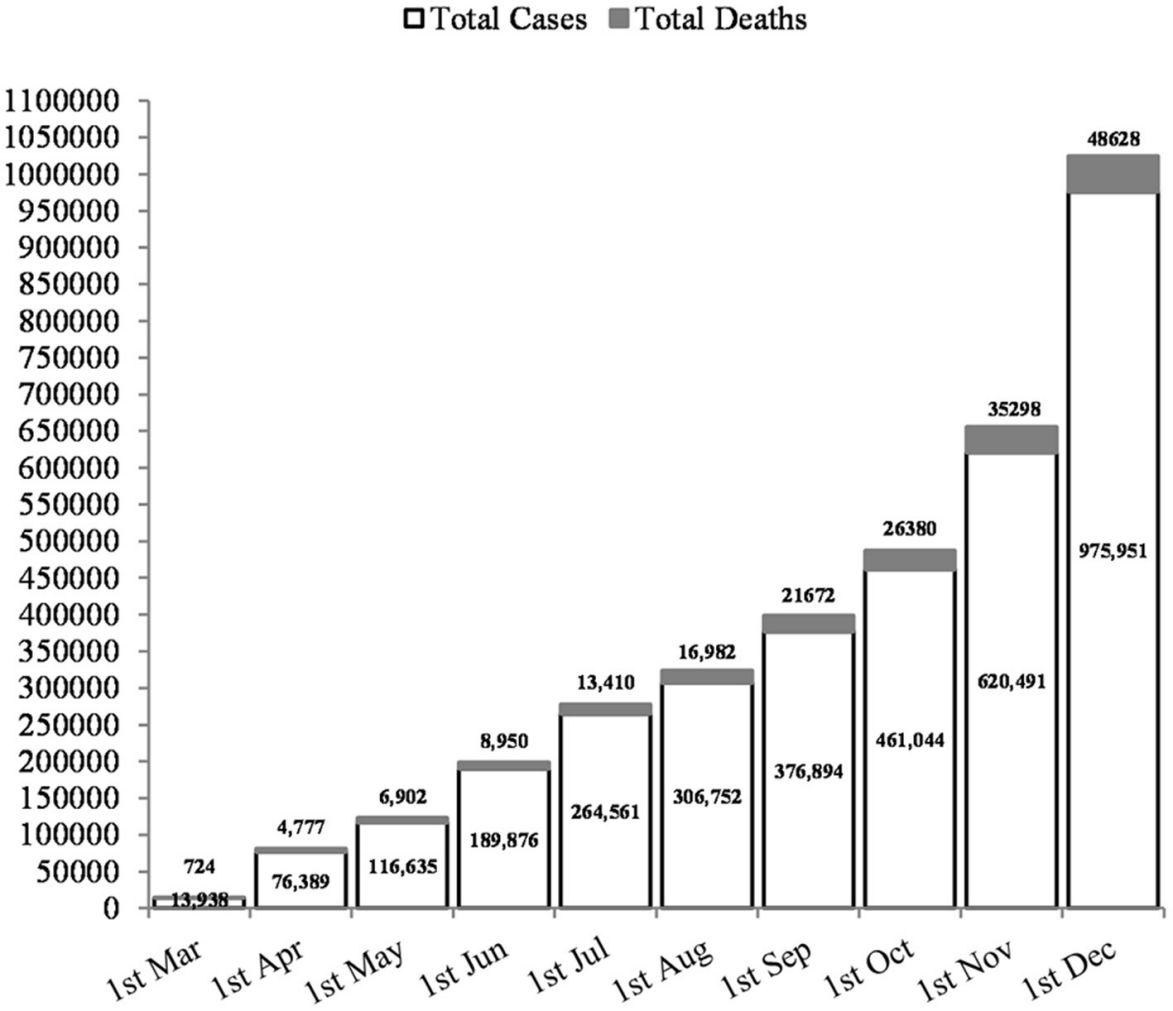
Rates of COVID-19 cases and deaths in Iran, as an Islamic country with the highest incidence.

Islamic countries have some of the most populous political and economic centers in the world with special religious and cultural practices, such as the annual Hajj of Mecca in Saudi Arabia, the pilgrimage of Imam Reza in Mashhad, and the consumption of unique animal foods such as camels (8, 9). About 2.5 million Muslims from Islamic countries travel to Mecca each year to perform the Umrah pilgrimage. Also, more than 20 million people visit Karbala in Iraq, another important Shia Muslim religious event, which goes on for 40 days. It has been reported that many pilgrims are hospitalized due to respiratory illness during these gatherings (10–12). One of the most important issues in recent research is that COVID-19 has a surface receptor on lung cells, esophageal epithelial cells, and ileum enterocytes: this is called the angiotensin converting enzyme 2 (ACE-2) (13, 14). It is noteworthy that the expression of this receptor is higher in people of Asian countries than in Europe and the United States, and also in men more than women (15, 16). In addition, many Islamic countries can be very vulnerable to COVID-19 due to their poor healthcare system and lack of facilities. The lack of facilities has probably led to many people with COVID-19 are not identified in Islamic countries, and the number of people infected with this dangerous virus is higher than reported. Currently, the World Health Organization (WHO) report has shown that, of all the Islamic countries infected with the COVID-19 virus, Iran has the highest incidence, with about 300,000 cases and 17,000 deaths (Figure 1) (17). The WHO website (https://www.who.int/emergencies/diseases/novel-coronavirus-2019/situation-reports) shows daily report of disease in all parts of the world.

With the spread of the deadly COVID-19 virus, there may be many questions such as whether fasting can be a predisposing factor to the COVID-19 virus? Does fasting impair the immune system? Does fasting reduce physical strength?

## Health-Related Effects of Ramadan Fasting

**Figure 2:**
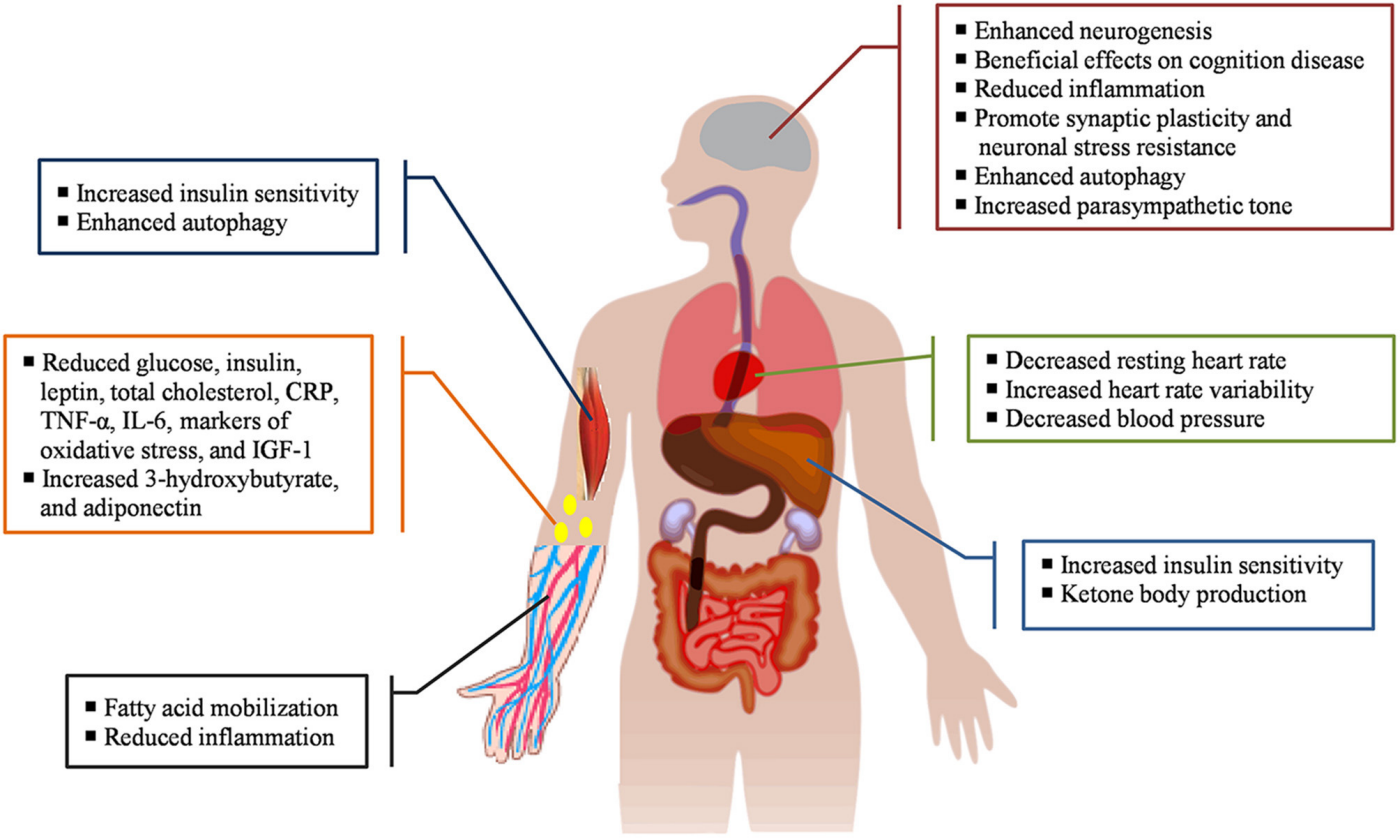
A summary of the main beneficial effects of fasting in different parts of the body.

Consumption of high-calorie foods leads to obesity and subsequent conditions of chronic inflammatory disease, which is known as low-grade inflammation (18, 19). Under these conditions, the observed concentration of inflammatory cytokines usually increases by 2 to 3 times. This leads to many autoimmune diseases such as rheumatoid arthritis and inflammatory diseases such as insulin resistance, atherosclerosis, tissue damage associated with different types of cancer, and cardiovascular disease (20). However, human and animal studies have shown that diets, especially those that mimic fasting, improve many health indicators, both in healthy people and in people with chronic diseases. Although the results can vary slightly depending on the type of fasting patterns and the species studied, all fasting-like diets can result in a fundamental metabolic change and may associate with some health outcomes (21, 22). It is interesting to note that, over the past few years, the beneficial effects of some types of fasting such as reduced meal frequency (diets with reduced meal frequencies such as every-other-day fasting), caloric restriction (typically involving a 15–40% reduction in daily energy intake with maintenance of nutrition), and alternate-day fasting (generally comprise 24-h periods of fasting alternating with 24 h periods of *ad libitum* feeding) have been shown to increase lifespan, improve insulin sensitivity, reduce oxidative stress and inflammation, as well as mortality of cancer and cardiovascular disease (23–27). However, major beneficial effects of Ramadan fasting on people's health (Figure 2) also will be discussed, together with the main mechanisms for improvement, in the following sentences (28):

1) It causes weight loss and maintenance among overweight and obese people (29).
2) Glucose homeostasis occurs amongst obese individuals with type 2 diabetes, together with reduction in percentage body fat and in HbA1c (29).
3) There is a greatly improved survival rate and recovery of heart function and modulate cardiovascular risks (30).
4) It may protect neurons against aging disorders (e.g., Alzheimer's disease and stroke) (31, 32).
5) Fasting reduces insulin resistance vs. continuous energy restriction amongst the overweight and, in obese, non-diabetic subjects, it may therefore have an important role in protecting against obesity-related cancers (33).
6) There are decreases in resting heart rate, insulin, circulating levels of glucose and homocysteine which are favorable with regards to the risk of cardiovascular disease (34, 35).
7) It reduces fat mass, total cholesterol, and LDL cholesterol (29).
8) There is equivalent reduction in blood pressure (35).
9) There is a reduction in serum triglycerides, markers of oxidative stress and inflammation (29).
10) Circulating ketone levels are also elevated on the fasting days (36).
11) Effects on a number of cancer risk biomarkers occur (e.g., insulin, cytokines, and the inflammation-related molecules leptin and adiponectin) which are thought to mediate the effects of adiposity and excessive energy intake on the development and growth of cancers in humans (35, 37).
12) A reduction in insulin-like growth factor 1 has been reported in normal and overweight subjects (38).

Overall, the abovementioned recent small trials of Ramadan fasting in multiple patients have been presented promising results that prepare the main reason for moving forward to perform larger clinical trials in patients with a wide range of disorders. However, this area of research is still in its infancy and requires the cooperation of various researchers and further research before reaching a definitive conclusion.

## Ramadan Fasting May Affect Immune Function

The immune system in the human body is an organization consisting of cells and molecules that play a role in defending against infections. The two types of immune system include the innate immune response, which acts the same when exposed to an infectious agent multiple times, and the acquired immune response, which is enhanced by repeated exposure to the infectious agent (39). In the innate immune response, phagocytic cells (macrophages, neutrophils, and monocytes), cells that secrete inflammatory mediators (eosinophils, mast cells, and basophils), and natural killer cells play key roles. Also, cytokines, complement, and acute phase proteins are molecular components of the innate immune system. But the cells involved in the acquired immune response are antigen-specific B and T cells, which proliferate when their surface receptors bind to antigens. B cells release antibodies that target extracellular microorganisms. The role of T cells in acquired immunity is to help B cells to producing antibodies and also kill virus-infected cells and intracellular pathogens by activating macrophages (39).

There have been many studies on the effects of Ramadan fasting on the immune system, which have shown that fasting can restore the immune system (20, 40). Fasting for at least 3 days allows the body to start producing new white blood cells, which rejuvenates the immune system to fight infection. Although it has been shown in humans and animals that the number of white blood cells decreases with long-term fasting, blood cells return when they are re-fed (40). In this way, Ramadan fasting mimicking diets for 3 days (intermittent fasting during Ramadan, time-restricted feeding, and alternate day fasting) forces the body to consume glucose and fat stores, and a significant amount of white blood cells is broken down. As a result, changes in the body cause the stem cells to regenerate new cells in the immune system (20, 41). The promising results of studies have shown that inflammatory cytokines (e.g., IL-1β, IL-6, and TNF-α), oxidative stress markers and C-reactive protein (CRP) might be reduced by Ramadan fasting (20, 42, 43).

On the other hand, various studies have confirmed that cytokine storms are an essential mechanism of coronaviruses, which produces large amounts of inflammatory cytokines such as IFN-α, IFN-γ, IL-1β, IL-6, IL-12, IL- 18, IL-33, TNF-α, G-CSF, IP-10, MCP-1, MIP-1A, TGF-β, as well as chemokines including CXCL9 and CXCL10 against the virus infection (44–46). The cytokine storm triggers an uncontrollable systemic inflammatory response in the patient that causes the immune system to attack the body and cause diseases such as respiratory failure, acute respiratory distress syndrome (ARDS), multiple organ failure, shock, and death (severe cases) (47, 48). Accordingly, the main mechanism of the COVID-19 virus is the inflammatory cytokine storm. However, Ramadan fasting in healthy people has been concluded to have a small impact on the markers of oxidative stress (protein carbonyls, 8-isoprostane, nitrotyrosine, and 4-hydroxynonenal adducts) and inflammation (serum IL-1β, IL-6, TNF-α, and C-reactive protein) (42). In the case of the COVID-19 pandemic, scientists, jurisprudential scholars, and physicians are not sure whether fasting is safe or not. On the other hand, in some Islamic schools, the fear and danger of infection are not considered a good justification for not fasting. Therefore, the final decision to Ramadan fasting in these circumstances should be made by each person (based on the fatwa) based on the recommendations of the responsible doctors (32). Here are some preventative suggestions for people who are eager to fasting under the COVID-19 pandemic conditions:

- Adherence to the WHO recommendations: this includes frequent hand washing; maintaining a distance of at least one meter from others, and wearing a mask.
- Most religious authorities have always stated that, if a person has problems with ill-health, it is better to refrain from fasting.
- During fasting, avoid being in a crowded public space such as bus, subway, etc.
- Have more rest during fasting.
- Avoid going to religious places for prayer and supplication (49, 50).

It has been proven that supporting immune function and enhancing individual resistance are essential to fighting COVID-19, and the most important ways to strengthen and boost personal immunity are to avoiding overconsumption of calories and to undertake proper exercise program (28, 51–53).

## Exercise Training, Ramadan Fasting and Immune Function

Based on available literature in the area of exercise immunology, it can be stated that, in general, regular moderate-intensity exercise training with a short-lasting duration (i.e., 45–60 min) has anti-inflammatory and antioxidant effects that improve immune function through enhancing the functional activity of tissue macrophages against pathogens, recirculation of neutrophils, anti-inflammatory cytokines, immature B cells, cytotoxic T cells, natural killer cells, and immunoglobulins such as immunoglobulin A (54–59). In particular, each acute exercise bout can induce these immunoenhancing effects that add up over time to strengthen immune defense. For instance, a brief session of moderate-intensity exercise induced significant alterations in the pattern of circulatory cytokines related to increased cellular immune function (60). However, vigorous exercise training for prolonged periods of time (i.e., more than 2 h) may cause immunodepression through induction of oxidative stress, inflammatory cytokine cascade, and muscle damage or fatigue. This can lead to acute inflammation and increased susceptibility to infection and, for example, to upper respiratory tract illness (URTI) (54, 61–63).

Exercise training can be considered as a real challenge for Ramadan-fasted individuals, especially during COVID-19 outbreaks. The inability to eat and drink for many hours before and during exercise bouts contributes to the possibility of reduced levels of endogenous fuel in parallel with dehydration which continue until the end of the workout (64, 65). Moreover, daytime sleepiness, and feelings of increased malaise as well as lethargy are the factors that may negatively affect Muslim athletes toward undesirable mood swings (64, 65). Therefore, Ramadan-fasted individuals may experience relatively higher levels of fatigue and perceived effort in response to the same amount of work or exercise training during the month of Ramadan when compared to the non-Ramadan period (65–67). Although most athletes can observe Ramadan with a small loss of physical performance, Ramadan intermittent fast (RIF) can be dangerous for individuals with type I diabetes mellitus, and for participants in ultra-endurance events, particularly under hot conditions (68). However, avoiding exercise training during Ramadan (i.e., 29–30 consecutive days) results in a regression of some important exercise-induced changes such as cardiovascular and resistance adaptations. Thus, maintaining what you have performed in the previous month, without considerable progress in your exercise routine, would be imperative during Ramadan (69).

Much of the Ramadan fasting literature that has evaluated immunological or antioxidant biomarkers has primarily focused on the effect of RIF on resting measures in elite or recreational athletes maintaining their usual high training loads (70–74). In elite athletes who continue to train intensely during Ramadan, RIF may lead to small but significant increases in plasma IL-6 concentration (74), serum CRP, immunoglobulin A and G, non-enzymatic antioxidants such as vitamin A, haptoglobin, and α1-antitrypsin, whereas circulating leukocyte numbers, prealbumin, and homocysteine remained relatively unchanged, and serum vitamin E decreased (70). In another study, RIF had no effect on serum CRP, urea, apoprotein A1, apoprotein B, and cortisol in well-trained middle-distance runners who continued their usual competitive training in Ramadan (74). Moreover, RIF may affect the diurnal variations in immune parameters in trained athletes so that leukocyte counts measured in the afternoon were significantly lower than pre-Ramadan, while in the morning CRP levels were lower than pre-Ramadan and cortisol was higher than afternoon (73).

With regard to resistance exercise, maintaining a hypertrophic training program (4 sessions per week, 4–6 resistance exercises per session, 4 sets with a load of 10 RM, 2–3 min rest intervals between exercises and sets) throughout Ramadan has been shown to have no effect on CRP, plasma thiobarbituric acid reactive substance (TBARS), and mean circulating leukocyte, neutrophil, lymphocyte, and monocyte counts. However, it has increased uric acid values, and total activities of superoxide dismutase (SOD) and glutathione peroxidase (GPX) in trained bodybuilders (71, 72).

It seems that, in general, high-load exercise training programs completed by experienced athletes during Ramadan produce a myriad of small fluctuations in inflammatory and antioxidant responses, most of which are minor, and within the normal clinical ranges. Future studies, however, should be carried out to support these findings and ascertain other immune-related aspects of RIF.

Another important aspect of research concerning exercise training during Ramadan is related to the effects of RIF on acute immune responses to an exercise bout (75–77), in which there is an apparent need for more studies and better understanding of the pattern of these responses. A brief maximal exercise (30-s Wingate test) in active young men has been shown to result in a significant increase in plasma concentration of IL-12, as a pro-inflammatory cytokine, during (the 1st and 4th weeks) and after Ramadan (3 weeks later). However, these changes during the 4th week were lower than that of the 1st week and after Ramadan (75). On the other hand, time-of-day affects antioxidant status and muscle inflammatory responses following exercise during Ramadan. In this context, one study examined the effects of RIF and time-of-day on biochemical responses of male athletes to an intermittent exercise test. The study reported that post-exercise levels of creatine kinase (CK), lactate dehydrogenase (LDH), aspartate aminotransferase (AST) and alanine aminotransferase (ALT) in the evening (5 P.M.) were higher than in the morning (7 A.M.) during the 2nd and 4th week of Ramadan, whereas total antioxidant status was better in the morning (76).

Despite the biochemical fluctuations mentioned which lead to changes in the hormonal, immune and antioxidant defense systems, there is no obvious scientific evidence to propose a significant increase in physiological stress or chronic systemic inflammation (70, 73, 76). Therefore, given the importance of exercise training to maintain proper immune function, especially during the COVID-19 pandemic, a modified training approach (e.g., home-based workouts) aiming to maintain physical fitness and body mass or avoiding detraining would seem worthwhile for the sedentary fasting people (Table 1). Nevertheless, for Muslim athletes, the same training load as that being undertaken immediately before Ramadan is proposed (69, 78). It should also be noted that it is best to do exercise sessions in the morning or after breaking the fast during the month of Ramadan to minimize performance degradation and exercise-induced inflammatory responses (76, 78, 79). We propose, however, that exercise session can be performed about 1 h after iftar (meal eaten after sunset) ensuring that only a light meal is taken.

**Table 1:** Exercise guidelines for the inactive or the less active fasting people during COVID-19 outbreaks in Ramadan.

**Type**	**Intensity**	**Duration**	**Frequency**
Warm-up	40–50% of HRmax	7–10 min	At least 3 and preferably 5 days per week
Aerobic training	50–75% of HRmax	20–30 min	
Stretching	Until the onset of pain in the muscle	6–10 s per limb/muscle	
Resistance training	50–70% of 1RM	20–30 min	
Cool-down	40–50% of HRmax	5 min	

*HRmax, Maximal heart rate; 1RM, 1-repetition maximum*.

## Nutritional Strategies for Recovery From Exercise Training in Ramadan

The homeostasis of nutritional metabolism has a vital role in maintaining cell survival and normal immunologic functions (80). It is suggested that along with exercise training, the nutritional approach should also be taken into account to provide acute nutritional needs, to optimize the body's recovery after each exercise bout and to help the stability of the immune system (54, 55, 78, 81). Indeed, finding a balance between exercise training load and nutrition is a challenging concern during Ramadan. Fasted individuals cannot ingest anything (e.g., carbohydrate, water, etc.) during and immediately following day-time exercise: this is likely to result in altered immunological responses to exercise bouts (70–73, 75, 76, 78). Nevertheless, night-time training can be a simple and logical way out of the problem because it allows athletes to stay relatively well-hydrated and to maintain blood glucose levels through the consumption of *ad libitum* drinking, so that recovery from exercise session can be effectively accomplished (78).

Sufficient intake of carbohydrates, protein substrates, polyphenols, minerals and vitamins may produce better outcomes regarding exercise recovery and efficient immune function (54, 55, 81). Therefore, in the situation of the COVID-19 pandemic, a well-balanced diet containing drinking water, carbohydrate-rich foods, grains, nuts, seeds, fruits, and vegetables is necessary to maintain viral protection and reduce exercise-induced inflammation (54, 55, 81). Fasted individuals should consume these nutritional items by a pattern of small frequent meals from the iftar to sahour (the last meal eaten before beginning the fast).

With regard to pre-exercise nutrition, it is proposed that a light meal containing at least 1 g of carbohydrate per kilogram of body mass (82) is suitable for the iftar meal to increase net carbohydrate availability during the subsequent exercise session and to reduce the risk of insulin increase and hypoglycemia after the onset of exercise. In addition, the exercise bout should be performed at least 1 h after the iftar meal.

Intended exercise duration discussed in the present article is about 60 min: this could enhance immune function (55) and is not associated with limitations of energy supply if pre-exercise carbohydrate ingestion has been given adequate attention (82). However, frequent rinsing of the mouth with a carbohydrate solution, such as carbohydrate-electrolyte sports drinks, appears to lead to better performance (83).

During early recovery from moderate-intensity exercise that is suggested in the paper cited below, the availability of dietary carbohydrate (e.g., moderate and high glycaemic index carbohydrate-rich foods and drinks) is imperative to provide high rates of muscle glycogen synthesis (82) and promote pro-inflammatory reactions within skeletal muscle (84). The 1st h after exercise session are the “golden times” for storing glycogen and it seems that body's capacity reaches maximum with a carbohydrate intake of about 1 g per kilogram of body mass per hour (85).

Beside carbohydrate, post-exercise protein ingestion has some benefits on immune responses to exercise and skeletal muscle recovery (82, 86). Fasted individuals can intake 20–25 g of high-quality protein soon after the exercise bout to provide adequate plasma essential amino acids which are important for many training adaptations through the synthesis of new protein structures (e.g., myofibrillar, sarcoplasmic and mitochondrial contents) during the recovery period (87, 88). Fast digesting proteins (e.g., whey component of milk) may be a good choice to increase the plasma leucine content after exercise (89). Table 2 summarizes, in general, the potential nutritional suggestions that may result in better exercise-induced outcomes during COVID-19 outbreaks in the month of Ramadan.

**Table 2:** Practical nutritional guidelines for adult athletes who are eager to fasting under the COVID-19 pandemic situation.

**Nutrient**	**Description**
Carbohydrate (82, 90)	• *Before exercise:* 1 g/kg of carbohydrate-rich foods such as pasta, rice and bread at the iftar meal (1 h prior to exercise) • *During exercise:* small amounts of carbohydrate-electrolyte drinks including mouth rinse • *After exercise:* 1.0–1.2 g/kg/h of carbohydrate sources such as potatoes and pasta
Protein (86, 90)	• *After exercise:* 20–25 g for young, and 40 g for elderly athletes
Fruits and vegetables (91)	• Frequent meals of citrus fruits, tree fruits, stone fruits, grapes, apples, most berries, strawberries, and bananas from the iftar to sahour meal • Onions, parsley, celery seed, oregano, soybeans, soy-based foods, and legumes can be used in the iftar and sahour meals
Vitamins (81, 92, 93)	• Vitamin C: 200 mg per day • Vitamin D: 2000 IU (50 μg) per day • Vitamin B_6_: 1.3 mg per day • Vitamin B_9_: 400 μg per day • Vitamin B_12_: 2.4 μg per day
Others (81, 93, 94)	• Zinc: 8–11 mg per day • Selenium: 400 μg per day • Omega-3 fatty acids: 250 mg per day of EPA + DHA

*EPA, Eicosapentaenoic acid; DHA, Docosahexaenoic acid*.

## Conclusions

COVID-19 is a serious respiratory disease that has become a deadly pandemic with a very rapid spread in countries around the world, especially in Islamic countries because of their poor healthcare system and lack of facilities. Whilst Ramadan fasting may seem to be a harmful challenge for Muslims in this situation, some health benefits have been proposed in humans (Figure 2). Nevertheless, improving immune function and increasing individual resistance are essential to help fight COVID-19 (28, 51, 52).

**Figure 3:**
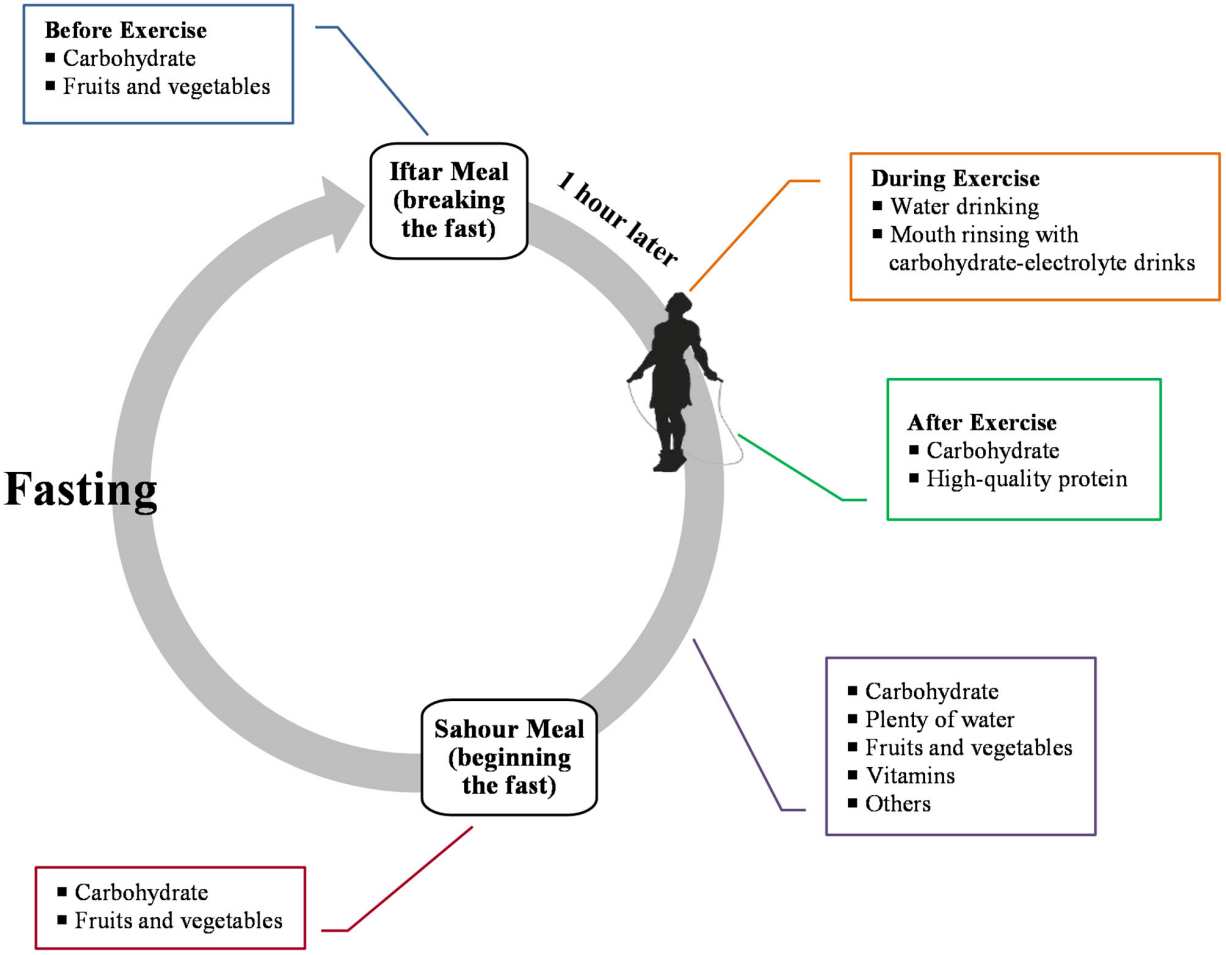
Timing of required nutrients during Ramadan with an emphasis on exercise training.

Exercise training has been known as a lifestyle factor that can maintain and even promote immune function and act like a vaccine against certain diseases/infections through producing physiological stress in the human body which lead to a series of adaptations occurring to overcome these stimuli (95). The overall health promotion effects of exercise training along with its disease prevention have resulted in the important statement of “Exercise is Medicine” (54, 96). These positive adaptations, however, may not be achieved without ingesting some functional foods (e.g., carbohydrate, protein, polyphenols, vitamins, and minerals) and fluids together with suitable timing (Figure 3) in order to help maintain exercise performance and immune function without causing harmful side effects on health (97).

## Author Contributions

MM and BT developed the idea, wrote, and drafted the paper. SP co-wrote and provided the figures and tables. KS co-wrote and developed the article. All authors have read and agreed to the published version of the manuscript.

## Conflict of Interest

The authors declare that the research was conducted in the absence of any commercial or financial relationships that could be construed as a potential conflict of interest.
